# Experiential Bloom’s Taxonomy learning framework for point-of-care diagnostics training of primary healthcare workers

**DOI:** 10.4102/ajlm.v5i1.449

**Published:** 2016-09-30

**Authors:** Tivani P. Mashamba-Thompson, Benn Sartorius, Fred C.J. Stevens, Paul K. Drain

**Affiliations:** 1 Department of Public Health Medicine, School of Nursing and Public Health, University of KwaZulu-Natal, Durban, South Africa; 2 Department of Educational Development & Research, Faculty of Health, Medicine and Life Sciences, Maastricht University, Maastricht, Netherlands; 3 Departments of Global Health, Medicine, and Epidemiology, University of Washington, Seattle, Washington, United States; 4 Department of Surgery, Massachusetts General Hospital, Harvard Medical School, Boston, Massachusetts, United States

## Introduction

The delivery of accessible, affordable and equitable primary healthcare (PHC) is a key focus in many resource-limited settings. One strategy that has been used to improve health access and healthcare equity in rural and resource-limited settings is the use of point-of-care (POC) diagnostics in PHC clinics. POC testing is defined as: pathology testing performed in a clinical setting at the time of patient consultation, generating a result that is used to make an immediate informed clinical decision that contributes to an improved health outcome for the patient.^1^ Although it relies on clinical, non-laboratory staff and frontline workers, such as nurses, to perform diagnostic testing, POC testing is seen as one of the ways to improve affordability, access and equity in rural areas. A primary advantage of POC diagnostics is that the completion of the test and treatment cycle in the same encounter is conducive to retention in care and patient outcomes.^2^

The advent of POC diagnostics in rural and resource-limited settings brings hope of improving health outcomes, particularly in HIV-epidemic countries. Prompt access to these diagnostics, in resource-limited settings with a high burden of disease, poor access to quality healthcare facilities and lack of laboratory infrastructure, is strongly encouraged.^3,4^ This will, however, demand task-shifting and the delegation of diagnostic services from laboratory-trained staff to non-laboratory-trained staff in PHC clinics. These changes could have serious consequences with regard to the quality of testing, especially as disease burden and, hence, testing volumes increase.

In this article, we discuss the significance of quality assurance as it pertains to POC diagnostic testing in PHC clinics. In addition, we suggest a training/retraining strategy for PHC nurses to ensure both the quality of POC diagnostic testing and the sustainability of quality control procedures in PHC clinics, by improving the proficiency and retention of skilled nurses.

### Concerns related to quality of point-of-care diagnostic services

The need for good quality assurance programmes to ensure accuracy of POC devices used to inform appropriate patient care in resource-limited settings has been demonstrated.^3^ Quality assurance programmes should include the following: running of internal quality control samples on every test day; regular calibration of the instruments; participation in external quality assessment schemes; adherence to standard operating procedures; and test operator competency checks.^5^

Research has also demonstrated poor regulatory control standards for the diagnosis of sexually-transmitted infections, in both public- and private-sector venues, within low- and middle-income countries.^6^ The entire process of POC diagnostics, from the pre-analytic through the analytic and post-analytic phases, has hidden reliability risks, including false-positive and false-negative test results, that can lead to gross medical errors. A false-positive result, such as an incorrect HIV-positive diagnosis in a person who is not infected with HIV, can lead to devastating consequences whereby patients may have to start antiretroviral treatment and change their lifestyle, which in turn may have a negative impact on their employment status and family relationships. A false-negative test result caused by a test’s failure to detect HIV antibodies or antigens in an HIV-infected person could lead to an HIV-negative diagnosis in an HIV-positive patient. This could have devastating consequences for the patient, particularly if their condition is at a stage where antiretroviral treatment is needed. It also increases the risk of infection of others, if the patient is sexually active.

Adequate PHC clinics must have the resources to enable appropriate storage and performance of diagnostic tests, as well as appropriate training of staff. The implementation of quality assurance practices is critical in ensuring the suitable administration of POC diagnostic tests and correct interpretation of their results.^7^ Inadequate human resources, due to high turnover rates of skilled nursing staff, are an ongoing problem in some high disease-burden countries such as South Africa, particularly at the rural PHC level.^8,9^ In addition, the competency of the diagnostic test user is one of the factors that affects the reliability of POC diagnostic services.^10^ It has been noted that there is a need for healthcare providers to be educated on the appropriate use of POC diagnostics, as well as a need for incorporation of POC diagnostics training into medical and nursing curricula as part of continual professional development.^11^

### Importance of competency of point-of-care diagnostic test users on service delivery

In recent years, there has been a significant increase in public- and private-sector investment in HIV and tuberculosis POC diagnostics.^12^ However, the complexity of the assay that can be used in a given setting is determined by the degree of infrastructure required for the assay platform.^13^ The competency of the user of POC diagnostic tests affects the reliability of the POC diagnostic services delivered in PHC clinics.^14,15^ Thus, the requirement not only for reliable electricity and climate-controlled testing rooms, but also for the staff skills and competencies required for more complex diagnostics, means that the majority of patients in resource-limited settings do not have access to the more complex assays at the community and PHC level.^14^ This potentially undermines the impact of POC diagnostics on patient outcomes.

### A strategy for improving the quality of primary healthcare clinic-based point-of-care diagnostic services

Despite the need for improving the availability of essential POC diagnostics in settings that lack laboratory infrastructure, such as rural PHC clinics,^3,4^ the shifting of the performance of diagnostic services to less-qualified and less-experienced staff could have serious consequences on the quality of testing, especially as disease burden and testing volumes increase. There is a need for a well-structured POC diagnostic training programme for all frontline health workers, particularly for those working in rural and resource-limited settings.

The overall aim of such a training programme would be to supplement current education and training resources for healthcare workers with a specialised POC diagnostics training curriculum. As stated above, since the competency of the healthcare professionals performing diagnostic POC testing influences the reliability of such services, we suggest a training strategy to equip frontline PHC workers with appropriate diagnostic knowledge and skills. This will help to ensure the quality, reliability and sustainability of POC diagnostic services.

Such training on the appropriate use of POC diagnostics needs to be incorporated into the medical and nursing curricula as part of continual professional development. In order to provide a real opportunity for ensuring the effectiveness and sustainability of such a programme, an appropriate educational strategy is essential. For example, a training programme that incorporates practical experiential learning is most desirable.

### Implementation of experiential learning for a point-of-care diagnostic training programme

Experiential learning, which is defined as a client-focused, supported learning approach,^16^ would help to engage users of POC diagnostics by inclusion of the following elements: action, reflection, and transfer to gain competency. We suggest incorporating a framework borrowed from Bloom’s taxonomy^17^ to guide the objectives of an experiential learning programme and to ensure quality of POC diagnostics services provided in PHC clinics (Table 1). This approach would provide PHC workers with a practical, cost-effective, on-site learning opportunity. Furthermore, it would enable direct exposure to POC diagnostic service delivery, rather than a traditional passive learning approach, which offers no exposure to service delivery.

**TABLE 1:** Objectives for point-of-care diagnostics experiential learning training programme for primary healthcare clinic workers using Bloom’s taxonomy.

Learning outcomes guided by Bloom’s taxonomy	Experiential learning objective	Intended outcome
Knowledge (5%)	A lecture and Q&A session on policies and principles for POC diagnostics services quality assurance.	Enhancing healthcare workers’ knowledge of POC diagnostics policies and principles to ensure quality service delivery.
	Practical training in delivery of PHC-based POC diagnostics services, which includes hands-on activities.	Concrete development of healthcare workers’ practical skills on application of POC diagnostics services.
Comprehension (25%)	Processing of new knowledge through reflection – reflective essay, with structured feedback, as part of a portfolio production exercise – producing evidence of learning and learner engagement.	Develop health worker’s skills, attitude and new ways of thinking about quality POC diagnostic service delivery.
Application (30%)	Work-based training – involving observation of POC diagnostic service delivery in a PHC setting.	To enhance health worker’s intellectual knowledge and skills in quality POC diagnostic service delivery.
Analysis (10%)	Putting emphasis on the knowledge of policies and procedures that guide POC diagnostic services. Allowing students to assess the quality of POC diagnostic services against relevant quality assurance standards and policies.	To enhance knowledge of POC diagnostic policies and procedures to ensure continual quality service delivery.
Synthesis (20%)	Contextualising the experience in the real world though delivery of a POC diagnostic service, under supervision in actual settings.	Integrating the learning experience into regular practice and application to inculcate competency in health workers in POC diagnostic service delivery.
Evaluation (10%)	POC expert-driven professional oversight for the trained PHC-based POC diagnostics users. This will involve formative and summative assessment of the health worker’s performance on POC diagnostic service delivery.	Ensuring retention of the acquired knowledge and skill by the trained health workers as well as continual quality POC diagnostic service delivery. Successful healthcare workers will be awarded a certificate of competency on PHC-based POC diagnostic services.

*Source*: Adapted from Beard & Wilson (2002)^16^ and Marzano & Kendall (2006)^17^

Q&A, question and answer; POC, point-of-care; PHC, primary healthcare.

However, due to the ever-changing nature of healthcare services and associated technologies, implementation of an experiential learning strategy alone will not guarantee continual quality service delivery and retention of skilled staff. Therefore, complementary strategies are needed, including continual mentorship, monitoring and evaluation to provide professional oversight to trained users of POC diagnostics.

### Professional oversight of staff trained to provide point-of-care diagnostic services

**FIGURE 1:**
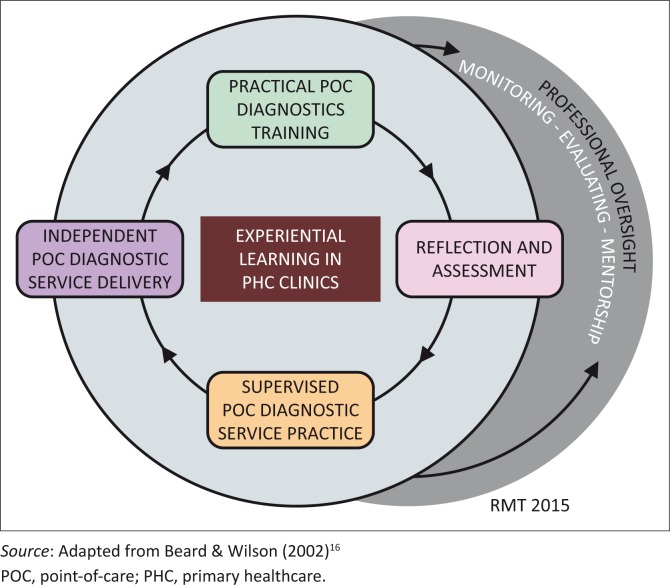
A Bloom’s taxonomy guided framework for implementing point-of-care diagnostics experiential learning programme to improve point-of-care testing proficiency and staff retention in primary healthcare.

To ensure the retention of the acquired knowledge and skills by the trained healthcare workers, we suggest an expert-driven process for professional oversight of trained PHC-based POC diagnostics users, using the following four strategies:^18^ (a) Regular monitoring of the learner’s competence on diagnostics through audits conducted by experienced POC diagnostic-competent healthcare workers with experience in PHC clinics; (b) Evaluation of the competency of trained POC diagnostics users by POC diagnostic-competent staff; (c) Retraining of previously-trained users with poor competency and new users replacing an existing user; and (d) Mentorship of trained POC diagnostics users by PHC clinic workers with competence and experience in POC diagnostics. Figure 1 depicts a framework for an experiential learning programme for continual, quality delivery of POC diagnostics services by PHC-based healthcare workers.

Appropriate implementation of the proposed programme will likely result in staff empowerment and provide an incentive to improve staff retention in PHC clinics. Successful implementation of the programme would require incorporation of a community-based, participatory research approach prior to and during implementation.^19^ This research approach would involve all stakeholders: researchers and healthcare workers, PHC clinic pathology service providers and policy makers. Such engagement should help invest stakeholders in team building and sharing of resources, open discussion and sharing of ideas and expertise to ensure continual quality delivery and effectiveness of the programme. An ‘enforcement’ approach to implementation is unlikely to achieve results.^20^ Rather, an approach developed through mutual consent and agreement is much more likely to be effective in the long term.^20^

## Conclusion

Delivery of high quality POC diagnostic services requires highly competent and dedicated staff. A special focus on well-structured POC diagnostic training strategies for improving staff competency in performance and quality maintenance for POC diagnostic services is urgently needed to ensure the highest quality diagnostic services. We recommend a guided framework, based on a Bloom’s taxonomy approach, for implementing a POC diagnostics experiential learning programme to improve POC testing proficiency and staff retention in PHC workers. Stakeholder engagement, consensus building and collaboration will be crucial to ensuring appropriate implementation and sustainability of the proposed programme.
